# Double mutation of cell wall proteins CspB and PBP1a increases secretion of the antibody Fab fragment from *Corynebacterium glutamicum*

**DOI:** 10.1186/1475-2859-13-56

**Published:** 2014-04-15

**Authors:** Yoshihiko Matsuda, Hiroshi Itaya, Yuki Kitahara, Natalia Maria Theresia, Ekaterina Aleksandrovna Kutukova, Yurgis Antanas Vladovich Yomantas, Masayo Date, Yoshimi Kikuchi, Masaaki Wachi

**Affiliations:** 1 Institute for Innovation, Ajinomoto Co., Inc, 1-1 Suzuki-cho, Kawasaki-ku, Kawasaki 210-8681, Japan; 2 Department of Bioengineering, Tokyo Institute of Technology, 4259 Nagatsuta, Midori-ku, Yokohama 226-8501, Japan; 3 Ajinomoto-Genetika Research Institute, 1st Dorozhny pr. 1, Moscow 113545, Russia

**Keywords:** CORYNEX®, *Corynebacterium glutamicum*, Protein secretion, Fab fragment, CspB, PBP1a

## Abstract

**Background:**

Among other advantages, recombinant antibody-binding fragments (Fabs) hold great clinical and commercial potential, owing to their efficient tissue penetration compared to that of full-length IgGs. Although production of recombinant Fab using microbial expression systems has been reported, yields of active Fab have not been satisfactory. We recently developed the *Corynebacterium glutamicum* protein expression system (CORYNEX®) and demonstrated improved yield and purity for some applications, although the system has not been applied to Fab production.

**Results:**

The Fab fragment of human anti-HER2 was successfully secreted by the CORYNEX® system using the conventional *C. glutamicum* strain YDK010, but the productivity was very low. To improve the secretion efficiency, we investigated the effects of deleting cell wall-related genes. Fab secretion was increased 5.2 times by deletion of *pbp1a*, encoding one of the penicillin-binding proteins (PBP1a), mediating cell wall peptidoglycan (PG) synthesis. However, this *Δpbp1a* mutation did not improve Fab secretion in the wild-type ATCC13869 strain. Because YDK010 carries a mutation in the *cspB* gene encoding a surface (S)-layer protein, we evaluated the effect of *ΔcspB* mutation on Fab secretion from ATCC13869. The *Δpbp1a* mutation showed a positive effect on Fab secretion only in combination with the *ΔcspB* mutation. The *ΔcspBΔpbp1a* double mutant showed much greater sensitivity to lysozyme than either single mutant or the wild-type strain, suggesting that these mutations reduced cell wall resistance to protein secretion.

**Conclusion:**

There are at least two crucial permeability barriers to Fab secretion in the cell surface structure of *C. glutamicum*, the PG layer, and the S-layer. The *ΔcspBΔpbp1a* double mutant allows efficient Fab production using the CORYNEX® system.

## Background

Recombinant antibody technologies can generate specialized whole antibodies or fragments with a myriad of potential therapeutic, diagnostic, and research applications [1]. Antibody fragments are particularly promising for clinical application because their ability to penetrate tumor cells is higher than full-length IgGs [2]. The fragment antigen-binding (Fab) molecule contains a fragmented heavy chain (HC) composed of the variable (V_H_) and the first constant (C_H1_) domains and a light chain (LC) composed of the light variable (V_L_) and constant (C_L_) domains. Production of recombinant Fab using microbial expression systems has been reported for several species, including *Escherichia coli*[3-8], *Pichia pastoris*[9-12], and *Saccharomyces cerevisiae*[13], but yields of active Fab have not been satisfactory.

*Corynebacterium glutamicum* is a Gram-positive, non-pathogenic soil bacterium [14,15] that has been used for industrial-scale production of amino acids such as glutamate and lysine for several decades [16,17]. *C. glutamicum* produces only small amounts of endogenous extracellular proteins compared with many other bacteria commonly used for protein production, a great advantage for protein purification. Thus, *C. glutamicum* is one of the most accessible and convenient bacterial species for biotechnology, but has not been used extensively for industrial production of proteins. We recently demonstrated that many heterologous proteins can be efficiently secreted in active form by the *C. glutamicum* ATCC13869 strain. Using a strong *cspB* promoter and signal peptides derived from a corynebacterial cell surface protein and the *Escherichia coli* twin-arginine translocation pathway, *C. glutamicum* ATCC13869 exhibited great potential as a host for industrial-scale production of recombinant proteins [18-24]. This protein expression system has been awarded trademark registration as CORYNEX®.

*Corynebacterium glutamicum* has a thick cell wall composed of two layers. The inner layer consists mainly of peptidoglycan (PG) and the outer layer mainly of mycolic acid. The presence of the outer layer may confer resistance against lytic enzymes, such as egg white lysozyme that catalyze hydrolysis of the β-1,4 glycosidic bond between the N-acetylglucosamine and N-acetylmuramic acid of PG [25], although this bacterium belongs to the Gram-positive category. This resistance is probably due to the function of the outer layer as a protein permeability barrier [26-29].

PG is synthesized on the outer surface of the cytoplasmic membrane by enzymes that bind to and are inhibited by β-lactam-type antibiotics such as penicillin (so that these enzymes are classified as penicillin-binding proteins, PBPs). In general, PBPs are membrane-bound proteins essential for cell wall synthesis by bacteria. They are classified into two types, high-molecular-weight PBPs (HMW-PBPs) and low-molecular-weight PBPs (LMW-PBPs). Further, HMW-PBPs are classified into class A HMW-PBPs having both a transpeptidase activity domain for crosslinking PG moieties and a transglycosylase activity domain for forming a polysaccharide chain, and class B HMW-PBPs having only a transpeptidase activity domain [30]. It is known that the class A HMW-PBPs of *C. glutamicum* are responsible for cell elongation, whereas the class B HMW-PBPs are responsible for formation of PG of septal walls at the time of cell division [30,31]. LMW-PBPs have D,D-carboxypeptidase activity and/or endopeptidase activity.

Several *C. glutamicum* strains have a surface (S)-layer outside the normal cell wall. The S-layer of many bacteria consists of a single protein assembled in two-dimensional paracrystalline arrays. The protein CspB (also called PS2) has been identified as a major secreted protein of several *C. glutamicum* strains [32,33] and forms the S-layer [34] in this species. Because of its location, the S-layer is generally involved in interactions between the bacterial cell and its environment. The S-layers of several pathogenic bacteria have been reported to act as virulence factors by conferring resistance to bactericidal activity [35,36] and by adhering to the extracellular matrix proteins of the host [37]. Furthermore, the S-layer can serve as a molecular sieve and act to stabilize the bacterial cell envelope of both pathogenic and non-pathogenic bacteria [38].

In the present study, we attempted to produce recombinant Fab using the *C. glutamicum* protein expression system CORYNEX®, but productivity was extremely low. We screened for mutations affecting the efficiency of Fab secretion and found that mutations in certain cell wall-related proteins enhanced Fab secretion, possibly by removing a physical and chemical barrier to secretion. This finding suggests that cell wall structures form a bottleneck for efficient recombinant Fab production in this expression system. The improved CORYNEX® system may enable industrial-scale Fab production.

## Results

### Secretion of antibody Fab fragments by *C. glutamicum* YDK010

**Figure 1:**
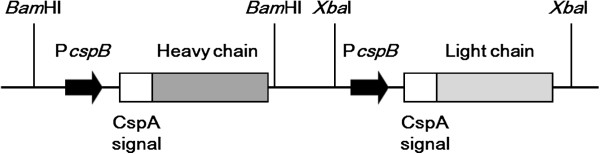
**Schematic diagram of the co-expression cassette of Fab(H+L) in pPKStrastFabHL.** The Fab(H+L) expression plasmid pPKStrastFabHL was constructed as described in Methods. The HC and LC genes of the anti-HER2 Fab fragment (shaded bars) fused with the signal peptide of CspA from *C. ammoniagenes* (open bars) were expressed under control of the *cspB* promoter from *C. glutamicum* (thick arrows).

**Figure 2:**
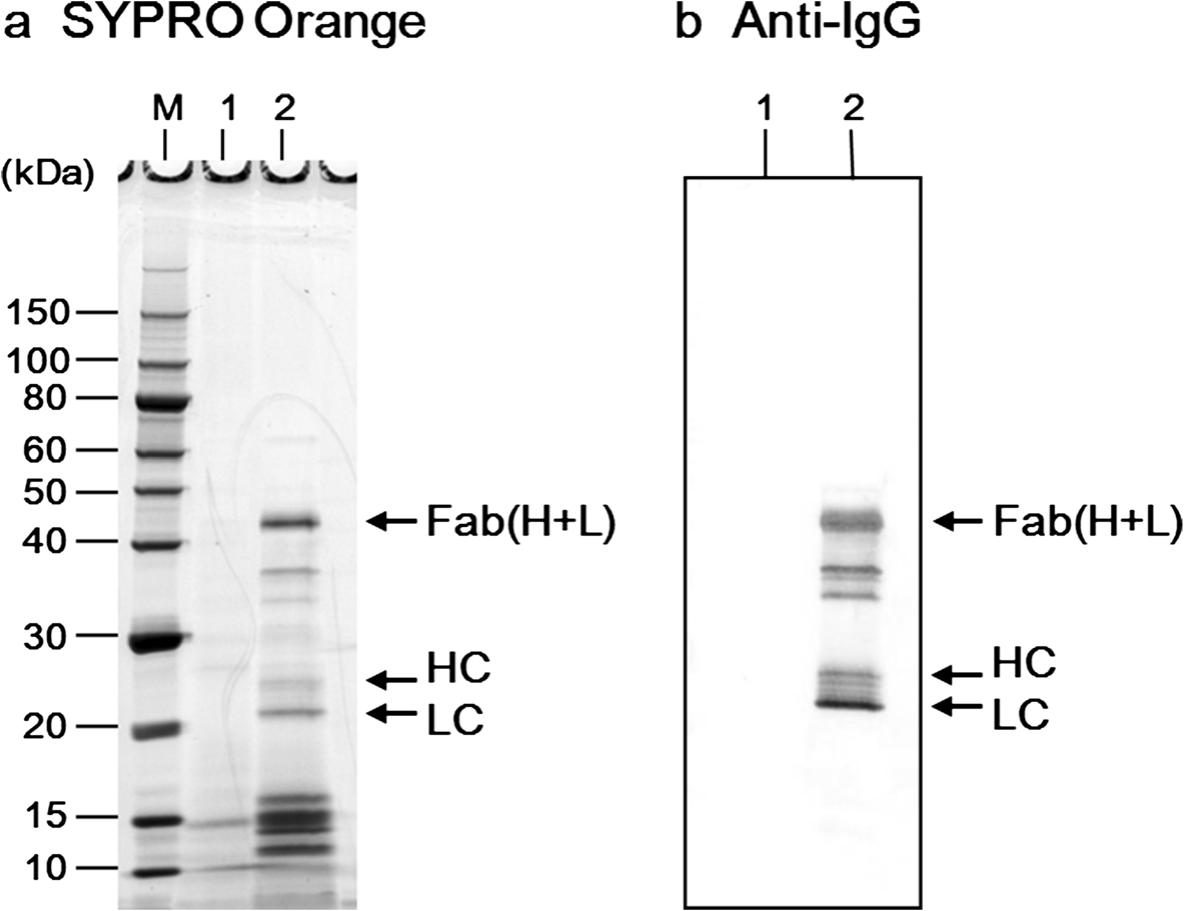
**Production of Fab(H+L) by *C. glutamicum *YDK010 carrying pPKStrastFabHL.** Supernatant proteins were separated by nonreducing SDS-PAGE. Ten microliters of supernatant mixed with an equal volume of sample buffer were loaded into each lane. **(a)** A gel stained with SYPRO Orange; **(b)** Western blot of supernatant proteins probed with anti-human IgG(H+L) antibody. Lane 1, YDK010/pPK4 (empty vector); lane 2, YDK010/pPKStrastFabHL. HC, heavy chain; LC, light chain; M, molecular weight marker.

Secretion of the Fab(H+L) fragment of the anti-HER2 antibody “trastuzumab”, used for targeted therapy of HER2^+^ breast cancer, was first assessed in the YDK010 strain. Bacteria were transformed with the pPKStrastFabHL plasmid containing HC and LC genes of the Fab region fused with the signal peptide derived from the *cspA* gene of *C. ammoniagenes* under the control of the *cspB* promoter of *C. glutamicum* (Figure 1 and Additional file 1: Figure S1). The transformant was then cultured in 4 ml of MMTG medium at 30°C for 96 h and the culture supernatant was analyzed by non-reducing SDS-PAGE and Western blotting with anti-human IgG(H+L) antibody. A secreted protein of about 45 kDa, corresponding to the molecular weight of Fab(H+L), was detected in the culture supernatant (Figure 2). This band was subjected to N-terminal amino acid sequencing, and, as expected, the N-terminal amino acid sequences of both HC (EVQLV) and LC (DIQMT) of the Fab(H+L) were detected. This result indicated that the signal peptide of CspA fused to both HC and LCs had been correctly processed for secretion and that HCs and LCs formed a heterodimer in the culture supernatant. However, accumulation of Fab(H+L) in the culture supernatant was barely detectable by Coomassie brilliant blue (CBB) staining. The secreted Fab yield was estimated to be approximately 11 mg/l. Protein bands of about 24 and 21 kDa, corresponding to the monomeric HCs and LCs, respectively, were also detected. Indeed, these bands had the expected N-terminal amino acid sequences of HC and LC. Other minor protein bands at 34–37 kDa were detected and may represent degradation products of Fab(H+L).

### Enhanced Fab secretion in the *pbp1a* deletion mutant *C. glutamicum* YDK010

**Figure 3:**
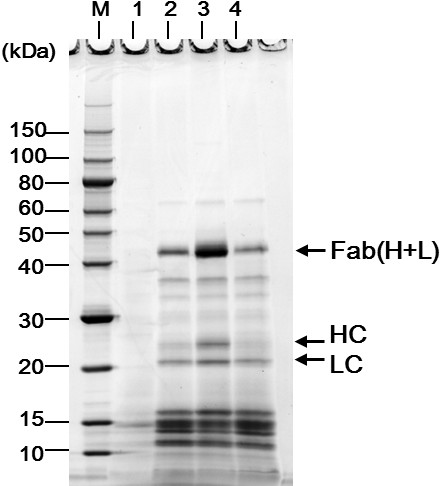
**Effect of *Δpbp1a *or *Δpbp1b *mutation on Fab secretion by *C. glutamicum *YDK010 strain.** Supernatant proteins were separated by non-reducing SDS-PAGE followed by SYPRO Orange staining. Ten microliters of supernatant mixed with an equal volume of sample buffer were loaded into each lane. Lane 1, YDK010/pPK4 (empty vector), lane 2, YDK010/pPKStrastFabHL; lane 3, YDK010*Δpbp1a*/pPKStrastFabHL; lane 4, YDK010*Δpbp1b*/pPKStrastFabHL; M, molecular weight marker. Mutation *Δpbp1a* but not *Δpbp1b* markedly increases Fab secretion from YDK010. The data shown represent three independent experiments that yielded similar results.

To improve the productivity of Fab(H+L) by the *C. glutamicum* YDK010 strain, we first investigated the effects of PBP gene deletion on the Fab secretion, as the PG layer synthesized by PBPs could function as a barrier to Fab secretion. Genome sequence data suggest that *C. glutamicum* has at least nine PBPs, of which the class A HMW-PBPs PBP1a and PBP1b and the class B HMW-PBPs PBP2a, PBP2b, and FtsI (PBP3) are major PG synthases [30]. Of these, only FtsI is essential for *C. glutamicum* growth [39], whereas the other four HMW-PBPs are dispensable [30]. The LMW-PBPs PBP4, PBP4b, PBP5, and PBP6 were characterized as carboxypeptidases and lactamases on the basis of sequence similarity analysis [30]. In this study, we investigated the effects of deleting each of these nonessential HMW-PBPs on Fab(H+L) secretion (Figure 3). Deletion of *pbp1a*, encoding the class A PBP1a, resulted in approximately 5.2 times higher secretion of Fab(H+L) than in the parent strain. In contrast, deletion of *pbp1b* had no effect on Fab(H+L) secretion. Similarly, deletion of *pbp2a* and *pbp2b* individually did not increase Fab(H+L) secretion (data not shown).

### Effect of *cspB* mutation on secretion of Fab(H+L)

**Figure 4:**
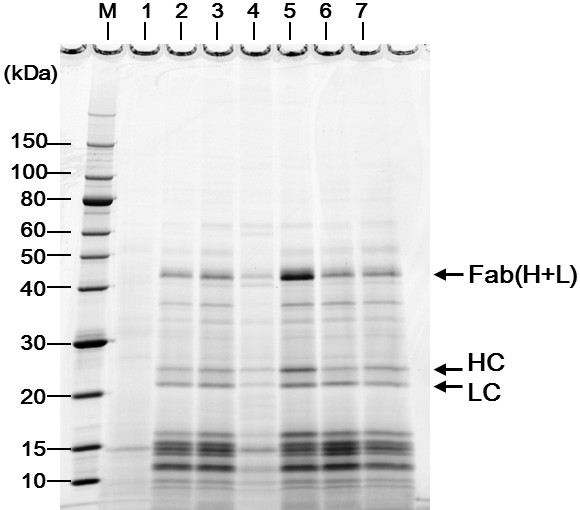
**Effects of *Δpbp1a *and *ΔcspB *mutations on Fab secretion by *C. glutamicum *wild-type strain ATCC13869.** Supernatant proteins were separated by nonreducing SDS-PAGE followed by SYPRO Orange staining. Ten microliters of supernatant mixed with an equal volume of sample buffer were loaded into each lane. Lane 1, ATCC13869/pPK4 (empty vector); lane 2, ATCC13869/pPKStrastFabHL; lane 3, ATCC13869*ΔcspB*/pPKStrastFabHL; lane 4, ATCC13869*Δpbp1a*/pPKStrastFabHL; lane 5, ATCC13869*ΔcspBΔpbp1a*/pPKStrastFabHL; lane 6, ATCC13869*Δpbp1b*/pPKStrastFabHL; lane 7, ATCC13869*ΔcspBΔpbp1b*/pPKStrastFabHL; M, molecular weight marker. The *Δpbp1a* mutation increases Fab secretion only in the presence of *ΔcspB* (*ΔcspBΔpbp1a* double mutant). The data shown represent three independent experiments that yielded similar results.

To confirm the positive effect of the *Δpbp1a* mutation on Fab(H+L) secretion, we evaluated the effect of *Δpbp1a* on the wild-type genetic background ATCC13869. Unexpectedly, *Δpbp1a* had no effect on Fab(H+L) secretion from ATCC13869 (Figure 4, lanes 2 and 4). It is known that during YDK010 strain construction, the *cspB* gene encoding the S-layer protein CspB is deleted [40]. We speculated that the *cspB* mutation was somehow involved in allowing Fab(H+L) secretion and investigated Fab secretion in both *ΔcspB* ATCC13869 [41] and the *ΔcspBΔpbp1a* double mutant ATCC13869. The *ΔcspB* single mutation only slightly affected Fab(H+L) secretion (Figure 4, lane 3), but the combined *ΔcspBΔpbp1a* mutation markedly enhanced Fab(H+L) secretion compared with both the single mutants and the wild type (Figure 4, lane 5). Thus, *Δpbp1a* increases Fab(H+L) secretion only in the presence of the *ΔcspB* mutation. In contrast, the *Δpbp1b* single mutation and the *ΔcspBΔpbp1b* double mutation did not affect Fab(H+L) secretion (Figure 4, lanes 6 and 7), coherent with the results from the experiment on YDK010 strain. The *Δpbp1a* mutation increased Fab secretion 5.2 times (11.1 mg/l → 57.6 mg/l) in the YDK010 strain and 3.9 times (8.9 mg/l → 34.9 mg/l) in the ATCC13869*ΔcspB* strain (Table 1). The YDK010 strain probably carries an unknown mutation that also affects the Fab secretion in the YDK010 background.

**Table 1:** Fab production yields by test tube culture

**Strain**	**Fab production yield (mg/l)**
YDK010	11.1 ± 0.3
YDK010*Δpbp1a*	57.6 ± 2.3
YDK010*Δpbp1b*	10.6 ± 0.5
ATCC13869	6.4 ± 0.4
ATCC13869*ΔcspB*	8.9 ± 0.8
ATCC13869*Δpbp1a*	2.2 ± 0.4
ATCC13869*ΔcspBΔpbp1a*	34.9 ± 1.8
ATCC13869*Δpbp1b*	8.1 ± 1.1
ATCC13869*ΔcspBΔpbp1b*	7.9 ± 1.3

The amount of secreted Fab in culture supernatant was quantified as described in Methods. Yields represent the average of measurements from three separate test tube cultures.

### Effect of *cspB* and *pbp1a* mutations on the lysozyme sensitivity of *C. glutamicum*

**Figure 5:**
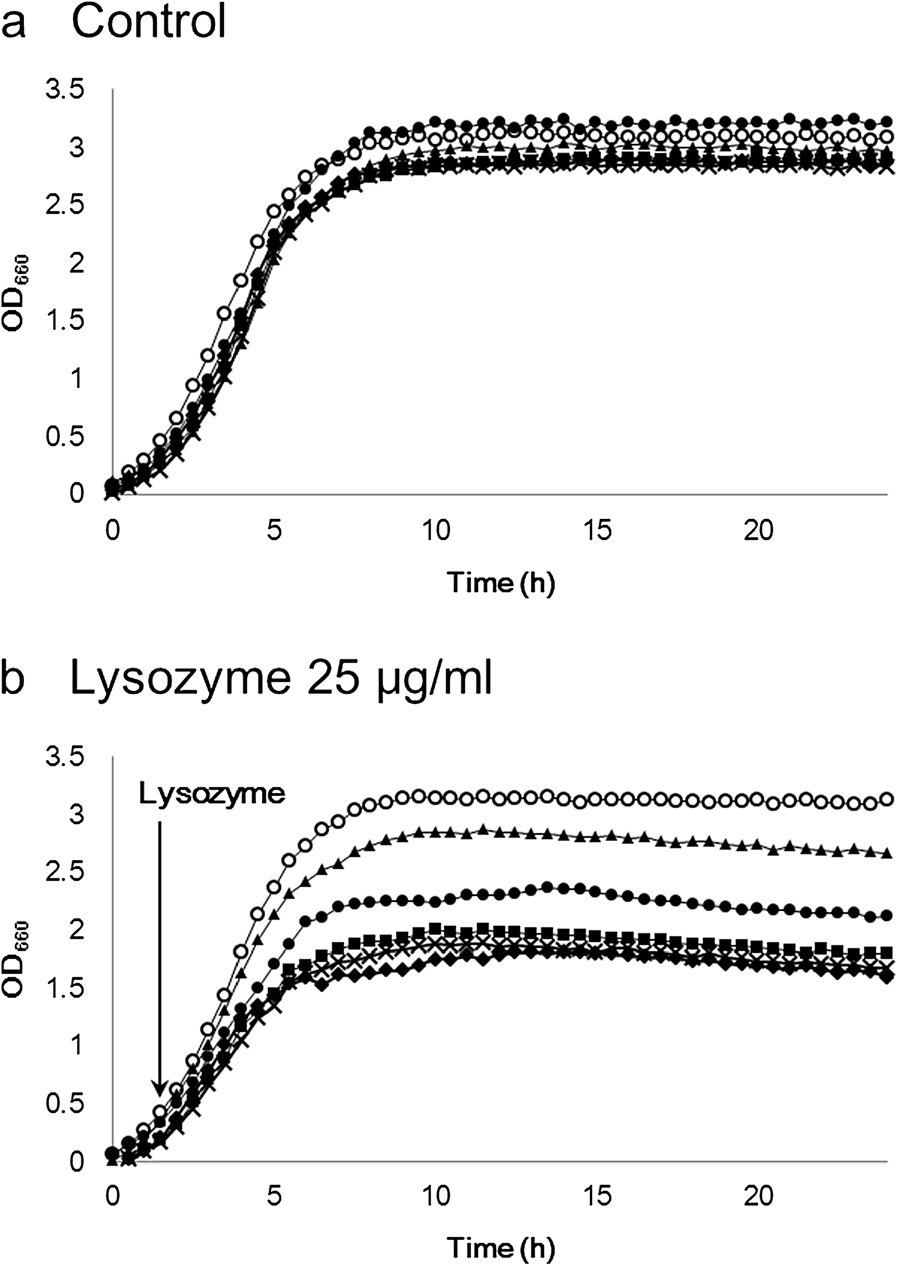
**Lysozyme sensitivity of *C. glutamicum *strains. (a) ***C. glutamicum* wild-type strain ATCC13869 (open circle), ATCC13869*Δpbp1a* (closed circle), ATCC13869*ΔcspB* (closed triangle), ATCC13869*ΔcspBΔpbp1a* (closed diamond), ATCC13869*Δpbp1b* (closed square), and ATCC13869*ΔcspBΔpbp1b* (X) were incubated in LB medium at 30°C. **(b)** Lysozyme (final concentration 25 μg/ml) was added to the cultures at the time indicated by an arrow. Growth was monitored by measuring OD_660_. The *ΔcspBΔpbp1a* double mutation considerably increased lysozyme sensitivity compared with either mutation alone or the wild-type strain. The *Δpbp1b* mutation showed higher lysozyme sensitivity than *Δpbp1a* mutation, but had no synergistic effect with *ΔcspB* mutation.

Both CspB and PBP1a are cell wall proteins, and thus, these mutations may affect cell surface integrity. To assess the effects on cell surface integrity, we examined the lysozyme sensitivity of each mutant strain, ATCC13869 WT, *ΔcspB*, *Δpbp1a*, *ΔcspBΔpbp1a*, *Δpbp1b*, and *ΔcspBΔpbp1b*, by growth assay in LB liquid medium. All strains exhibited similar growth rates under control conditions (Figure 5a). When 25 μg/ml lysozyme was added to exponentially growing cultures, growth of the wild-type strain was not affected, indicating strong lysozyme resistance (Figure 5b). In contrast, the growth rates of both *ΔcspB* and *Δpbp1a* single mutants decreased gradually during lysozyme treatment, resulting in lower final growth yields. The *ΔcspBΔpbp1a* double mutant showed higher sensitivity to lysozyme than did the *Δpbp1a* and *ΔcspB* single mutants (Figure 5b). These results suggest that the *ΔcspB* and *Δpbp1a* mutations affect cell surface integrity and that the two mutations act together to disrupt cell surface integrity and enhance lysozyme sensitivity. In contrast, the *Δpbp1b* single mutant showed higher lysozyme sensitivity than the *Δpbp1a* mutant, which was comparable to the *ΔcspBΔpbp1b* double mutant. However, no synergistic effect was observed between *ΔcspB* and *Δpbp1b* mutations.

### Antigen-binding activity of Fab secreted by *C. glutamicum*

**Figure 6:**
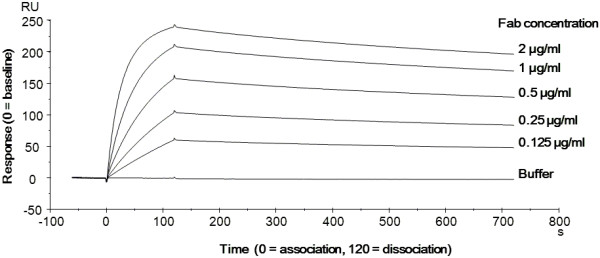
**Antigen-binding activity of Fab secreted by *C. glutamicum*.** The secreted Fab was partially purified and assayed for antigen-binding activity by Biacore X100. A CM5 sensor chip immobilized with the extracellular domain of recombinant human HER2/ErbB2 (577 RU) was used to obtain the binding sensorgram of Fab.

To determine whether the secreted Fab has a correct antigen-binding activity, it was partially purified from culture supernatant by protein G affinity column, and binding activity to its antigen (human HER2/ErbB2) was evaluated by surface plasmon resonance assay as described in Methods. As shown in Figure 6, the Fab secreted by *C. glutamicum* had binding affinity to the antigen. The dissociation constant (*K*_D_) value of this partially purified Fab was calculated to be 0.36 nM, a value comparable to that in a previous report by Khalili, et al. [42]. This confirmed that the secreted HC and LC assembled each other and formed a complete Fab structure in the supernatant.

## Discussion

In this study, we attempted to produce the clinically important antibody Fab fragment, a heterodimeric molecule containing an intermolecular disulfide bond, using the CORYNEX® system. As shown in Figure 2, the recombinant Fab(H+L) was secreted into the culture medium by the industrial strain YDK010, in addition to the monomeric HC and LC. Sequencing of the N-terminal amino acids revealed that the signal peptide derived from *C. ammoniagenes* CspA was properly cleaved during secretion. It was also shown that an intermolecular disulfide bond was formed between HC and LC. The secreted and partially purified Fab showed high binding activity to its antigen HER2/ ErbB2 (Figure 6). This is the first Fab expression experiment performed in *C. glutamicum* by the conventional CORYNEX® system using the *cspB* promoter and the Sec-dependent CspA signal peptide. Recently, secretion of antibody single-chain variable fragment (scFv) in *C. glutamicum* was also reported [43-45]. Yim et al. reported the secretion of scFv in the *C. glutamicum* ATCC13032 strain, with approximately 18 mg/l in flask cultivation and 68 mg/l in a 5-L bioreactor, using a fully synthetic H36 promoter and the Sec-dependent PorB signal peptide [45]. scFv is a monomeric molecule, whereas Fab is a heterodimer containing an intermolecular disulfide bond. The present report thus reveals the ability of *C. glutamicum* to secrete a recombinant heteromultimeric protein in active form. Production of recombinant antibody fragments using microbial expression systems has also been reported, with yields of several dozen mg/l [3-13]. In our study, *C. glutamicum* showed the ability to secrete recombinant antibody fragment at equal or even higher levels than other expression systems. Moreover, this secretion system has a great advantage for protein purification because *C. glutamicum* produces only small amounts of endogenous extracellular proteins. However, accumulation of the secreted Fab in the YDK010 strain was still low. To identify the bottleneck in Fab production in *C. glutamicum*, we investigated the effects of specific cell wall-related genes and succeeded in developing a strain with improved secretion ability by mutating *cspB* and *pbp1a*.

We first screened the nonessential PBPs in the industrial strain YDK010 (originally derived from the wild-type ATCC13869 strain) and found that the *Δpbp1a* deletion markedly improved Fab secretion (Figure 3). However, this effect was not observed on the wild-type background. Further genetic analysis revealed that a mutation in the *cspB* gene was also involved in the improvement of Fab secretion. A reconstituted double mutant *ΔcspBΔpbp1a* on the wild-type ATCC13869 background, but neither of the constituent mutations alone, showed improved Fab secretion (Figure 4). Both *cspB* and *pbp1a* are cell wall-related genes; PBP1a is involved in PG synthesis, and CspB forms the cell wall S-layer. These results suggest that there are at least two crucial permeability barriers to Fab secretion in the cell surface structure of *C. glutamicum*.

Because these two genes are both cell wall-related genes, mutations in them may affect the integrity of the cell surface structure. Indeed, a lysozyme sensitivity test supported this idea. Both the *ΔcspB* and *Δpbp1a* single mutant strains showed higher lysozyme sensitivity than the wild type, whereas the *ΔcspBΔpbp1a* double mutant strain showed far greater sensitivity than either single mutant strain (Figure 5). These results indicate that these mutations affect cell surface integrity. Lysozyme sensitivity could be an effective parameter for screening mutations (such as *cspB* and *pbp1a*) that affect, either directly or in combination with other mutations, protein secretion in *C. glutamicum*.

CspB is a structural protein of the S-layer that forms solid two-dimensional paracrystalline arrays surrounding the entire cell [37]. It is likely that the S-layer interferes with the release of Fab into the extracellular space. Genome sequence data suggest that *C. glutamicum* has at least nine PBPs [30]. Mutants of *pbp1a* and *pbp1b*, encoding class A HMW-PBPs, exhibited a similar morphological phenotype [30], although these proteins have distinct binding partners [31]. PBP1a interacts with the cell division protein DivIVA, whereas PBP1b interacts with the morphogenic protein RodA [31]. PBP1b also interacts with PBP2a and PBP2b, though PBP1a does not interact directly with any other HMW-PBP [31]. Thus, PBP1a may have a distinct function among HMW-PBPs in *C. glutamicum*, possibly related to protein secretion.

*C. glutamicum* has a (coryno)mycolate hydrophobic layer between the PG-arabinogalactan layer and S-layer thought to function as a permeability barrier to antibiotics and host defense molecules [26-29]. It is still unclear how secreted proteins pass through this hydrophobic layer. The mycolate layer is composed of free trehalose mycolates and mycolic acid covalently bound to arabinogalactan, which is in turn attached to the PG layer [28,29]. Loss of PBP1a may affect formation of the mycolate layer by modifying the PG-arabinogalactan layer.

We also examined the secretion of proteins other than Fab, but the *ΔcspBΔpbp1a* double mutation was only effective for Fab as far as tested (data not shown). This suggests that the bottleneck in protein production is different in each case.

In addition to Fab and constituent monomers, protein bands at approximately 34–37 kDa were detected in the culture supernatant of Fab producer strains. It is clear that these were the degradation products of the secreted Fab, because they were specifically detected by Western blotting with anti-IgG (Figure 2b). Protein bands of approximately 12–16 kDa were also detected by SYPRO Orange staining and did not react with anti-IgG. These could be degradation products of Fab, because they were not detected in the culture supernatant of the strain carrying the empty vector pPK4 (Figure 2a). The protease(s) responsible for Fab degradation are unknown. Identification and deletion of such protease(s) is expected to further improve Fab production by the CORYNEX® system.

## Conclusions

**Figure 7:**
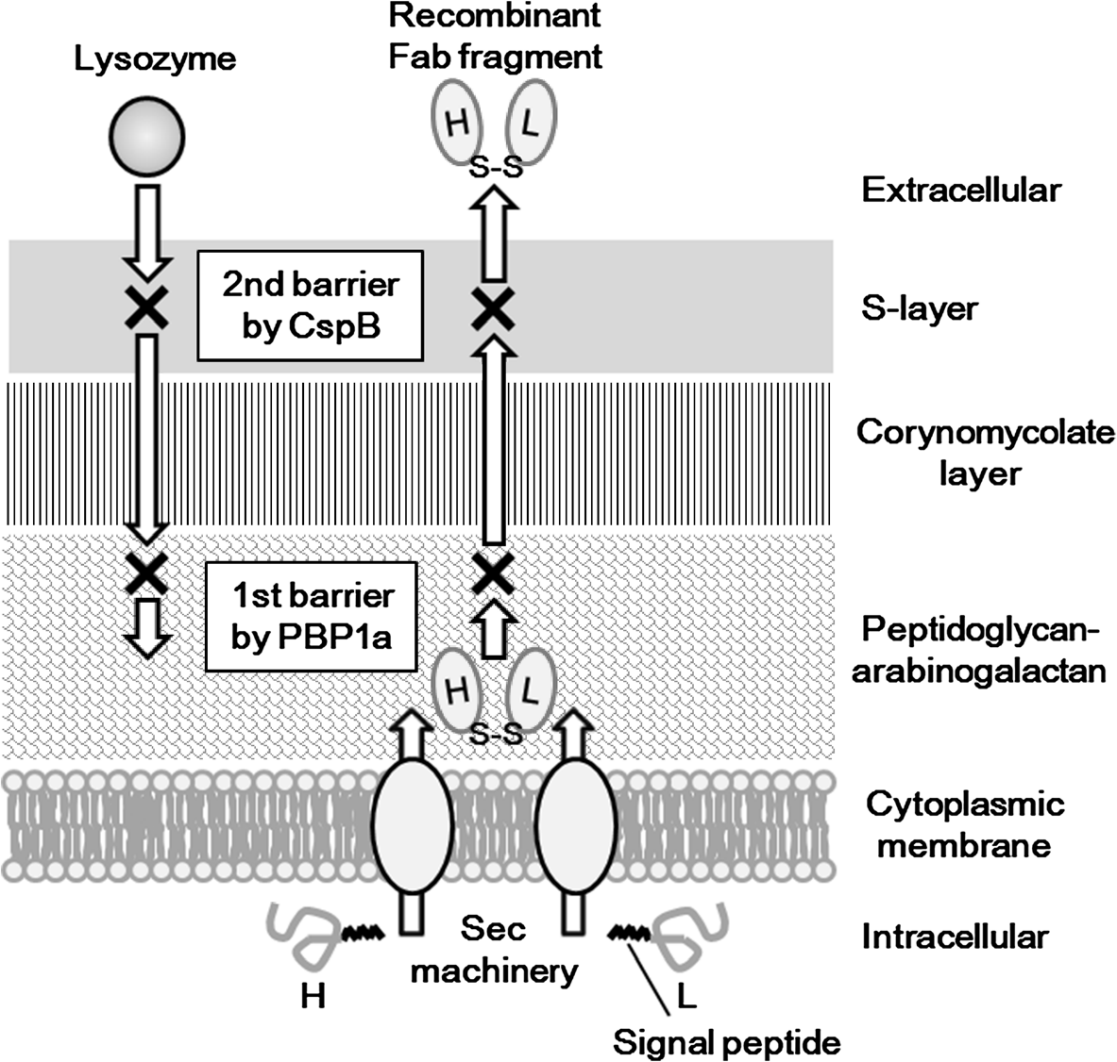
**Cell wall-permeability barriers to the recombinant Fab(H+L) secretion by *C. glutamicum*.** HC and LC of the Fab fragment are secreted through the cytoplasmic membrane by the Sec machinery and form a heterodimer with an intermolecular disulfide bond (S–S) in the extracellular space. There are at least two crucial permeability barriers (X) interrupting Fab secretion; the PG-arabinogalactan layer synthesized by PBP1a and the S-layer composed of CspB. These two structures also function as permeability barriers to lysozyme.

We have shown that a combination of *ΔcspB* and *Δpbp1a* mutations improves recombinant Fab secretion from *C. glutamicum*, suggesting that there are at least two permeability barriers to Fab secretion in *C. glutamicum*: the PG layer and the S-layer (Figure 7). Cell wall-associated genes are thus promising targets for further improvement in recombinant protein secretion by *C. glutamicum*. Moreover, lysozyme sensitivity could be an effective parameter for screening mutations that affect protein secretion.

## Methods

### Bacterial strains, plasmids, and culture media

The bacterial strains and plasmids used in this study are listed in Additional file 2: Table S1. *E. coli* JM109 was grown in Luria–Bertani (LB) broth [46] and used for plasmid construction. *C. glutamicum* wild-type strain ATCC13869 and its derivative YDK010, an industrial strain for protein secretion [40], were used. *C. glutamicum* strains were grown at 30°C in modified CM2G medium [18] consisting of 10 g/l polypeptone, 10 g/l yeast extract, 5 g/l glucose, 5 g/l NaCl, and 0.2 g/l DL-methionine (pH 7.2), or modified CMDex medium [41] consisting of 5 g/l glucose, 10 g/l polypeptone, 10 g/l yeast extract, 1 g/l KH_2_PO_4_, 0.4 g/l MgSO_4_ · 7H_2_O, 3 g/l urea, 0.01 g/l FeSO_4_ · 7H_2_O, 0.01 g/l MnSO_4_ · 5H_2_O, 1.2 g/l (as total nitrogen) soybean hydrolysate, and 10 μg/l biotin (pH 7.5). For antibody Fab fragment secretion, *C. glutamicum* strains were cultured in 3 ml of liquid CM2G medium at 30°C overnight and 0.2 ml of the cultures were inoculated in 4 ml of modified liquid MMTG medium [18] consisting of 120 g/l glucose, 3 g/l MgSO_4_ · 7H_2_O, 30 g/l (NH_4_)_2_SO_4_, 1.5 g/l KH_2_PO_4_, 0.03 g/l FeSO_4_ · 7H_2_O, 0.03 g/l MnSO_4_ · 5H_2_O, 0.45 mg/l thiamine hydrochloride, 0.45 mg/l biotin, 0.15 mg/l DL-methionine, 0.2 g/l (as total nitrogen) soybean hydrolysate, and 50 g/l CaCO_3_ (pH 7.0) in a test tube, and then cultured at 30°C for 96 h. Kanamycin (25 μg/ml) was added to the culture medium as required. To prepare agar plates, agar (20 g/l) was added to the growth media.

### Construction of a plasmid for Fab secretion

The DNA sequences encoding the variable regions of the HC and LCs of anti-HER2 (GenBank accession numbers, AY513484 and AY513485, respectively) and each constant region were designed by incorporating the *C. glutamicum* codon bias (GenScript, Piscataway, NJ, USA). DNA fragments containing the promoter of *cspB* from *C. glutamicum* and the signal sequence of CspA from *C. ammoniagenes* were fused to the HC or LC genes by GenScript to produce the tandem expression cassette (Figure 1 and Additional file 1: Figure S1). The synthesized construct was digested with *Bam*HI or *Xba*I, respectively, and inserted into the *Bam*HI and *Xba*I sites of pPK4 [18] to obtain pPKStrastFabHL. The cloned fragments were sequenced to confirm the intended construction.

### Protein analysis

The culture supernatant was obtained by centrifugation and 10 μl of supernatant was mixed with an equal volume of SDS sample buffer (Bio-Rad, Hercules, CA, USA) without reducing agent. Proteins were separated by 10%–20% gradient polyacrylamide gel electrophoresis (PAGE) as described by Laemmli [47] under non-reducing conditions, and the gels were stained with SYPRO Orange (Life Technologies, Carlsbad, CA, USA). Estimation of Fab production yield was done by quantifying the intensity of protein bands in gels using Multi Gauge software (Fujifilm, Tokyo, Japan). A standard curve was generated using a standard protein solution of known concentration run in the same gel. For determining the N-terminal amino acid sequence, proteins were transferred to a polyvinylidene difluoride (PVDF) membrane by electroblotting after separation by sodium dodecyl sulfate (SDS)-PAGE, and the protein bands were directly applied to a gas-phase protein sequencer (model PSQ; Shimadzu, Kyoto, Japan) equipped with an in-line amino acid analyzer (model RF-550; Shimadzu), as described previously [48]. Western blotting analysis was performed with alkaline phosphatase (AP) conjugated anti-human IgG(H+L) antibody (Rockland Immunochemicals, Gilbertsville, PA, USA) and an AP conjugate substrate kit (Bio-Rad, Hercules, CA, USA).

### Antigen-binding activity analysis of Fab

Culture supernatant was collected by centrifugation and filtered with a 0.22 μm Millex-GV syringe filter unit (Merck Millipore, Billerica, MA, USA). 20 ml of the filtrated supernatant was injected directly onto 1 ml of HiTrap Protein G affinity column (GE Healthcare UK. Ltd., Buckinghamshire, England) which was pre-equilibrated with 20 mM Tris–HCl (pH 7.5). Subsequently, Fab trapped in the column was eluted with 3 ml of 0.1 M Glycine-HCl (pH 2.7), and 20 μl of 2 M Tris–HCl (pH 8.5) was added immediately to bring to physiological pH. Biacore X100 (GE Healthcare UK. Ltd., Buckinghamshire, England) was used for surface plasmon resonance analysis. The extracellular domain of recombinant human HER2/ErbB2 (Sino Biological Inc., Beijing, China) was diluted in 10 mM sodium phosphate buffer (pH 6.0) and immobilized on a CM5 sensor chip (GE Healthcare UK. Ltd., Buckinghamshire, England) to achieve 577 resonance units (RU) by amine coupling according to the manufacturer’s instructions. Various concentrations of samples were injected into the flow cell diluted with HBS-EP buffer (10 mM HEPES pH 7.4, 150 mM NaCl, 3 mM EDTA and 0.005% surfactant P20). All kinetic measurements were conducted at 25°C at a flow rate of 30 μl/min with an association time of 120 s and dissociation time of 600 s. Chip regeneration was accomplished by exposing the chip to 10 mM Glycine-HCl (pH 1.5) for 120 s. Data were calculated using Biacore X100 evaluation software (GE Healthcare UK. Ltd., Buckinghamshire, England) by fitting the data to a 1:1 binding model.

### Construction of *pbp1a* and *pbp1b* deletion mutants of *C. glutamicum*

*C. glutamicum* disruptants were constructed as described previously [41]. Plasmid pBS5T [49], which carries a temperature-sensitive replication origin and the *Bacillus subtilis sacB* gene, was used as a suicide vector [50]. To construct a *pbp1a* disruptant, two successive rounds of PCR were performed. In the first-round, a 1.0-kb upstream region of the *pbp1a* gene was PCR-amplified from ATCC13869 chromosomal DNA template using the primers 5′-GTCGGATCCGCCCCCCTGAGCCAAATATTC-3′ and 5′-TTTCTAGCGGAAGAACTGGTTGATGGCGTCGAGCTTTGTCAGAGA-ATTCGTGGT-3′, and a 1.0-kb downstream region of the *pbp1a* gene was PCR-amplified using primers 5′- GTGTCCACCACGAATTCTCTGACAAAGC-TCGACGCCATCAACCAGTTCTTCC-3′ and 5′-AGTATCTAGATTCGAGTCGCTT-TTGGTTGGC-3′. Second-round PCRs were performed on the first-round PCR products using the primers 5′-GTCGGATCCGCCCCCCTGAGCCAAATATTC-3′ and 5′- AGTATCTAGATTCGAGTCGCTTTTGGTTGGC-3′. The amplified fragments were digested with *Bam*HI and *Xba*I and inserted into the *Bam*HI–*Xba*I site of pBS5T to obtain pBS5TΔpbp1a. Similarly, to construct a *pbp1b* disruptant, a 1.3-kb upstream region of the *pbp1b* gene was PCR-amplified from ATCC13869 chromosomal DNA template using the primers 5′-CGGCGAACTCAAAAACAGCAT-3′ and 5′-GGATAGTCAGCCCCGGCAGGATCCTTTTGCCACTGCTCTTTTTG −3′, and a 1.1-kb downstream region of the *pbp1b* gene was PCR-amplified using primers 5′- CAAAAAGAGCAGTGGCAAAAGGATCCTGCCGGGGCTGACTATC-3′ and 5′- CCAAACAACCCGAAGCTCAAC-3′ as first-round PCRs. Second-round PCRs were performed on the first-round PCR products using primers 5′-CGGCGAACTCA-AAAACAGCAT-3′ and 5′-CCAAACAACCCGAAGCTCAAC-3′. Amplified fragments were digested with *Pst*I and *Sal*I, and the resulting 2.2-kb fragment was inserted into the *Pst*I–*Sal*I site of pBS5T to give pBS5TΔpbp1b. Vector pBS5TΔpbp1a or pBS5TΔpbp1b was introduced into *C. glutamicum* by electroporation, and kanamycin-resistant transformants were selected at 34°C. Because pBS5T does not replicate at 34°C, only single-crossover chromosomal integrants grew on kanamycin-containing CMDex plates at 34°C. One of the kanamycin-resistant transformants was grown in CMDex medium without kanamycin overnight, and the cells were spread on sucrose-containing CMDex agar plates (10% sucrose). Cells carrying the *sacB* gene do not grow in the presence of sucrose, and thus, only cells in which the *sacB* gene was excised from the chromosome by a second homologous recombination event grew on the sucrose-containing plates. The resulting sucrose-resistant recombinants presumably had the wild-type or the deleted *pbp1a* or *pbp1b* gene, depending on the recombination points. The desired disruptants were selected by PCR. The *pbp1a* or *pbp1b* deletion mutants of *C. glutamicum* were designated as YDK010*Δpbp1a*, YDK010*Δpbp1b*, ATCC13869*Δpbp1a*, ATCC13869*Δpbp1b*, ATCC13869*ΔcspBΔpbp1a*, and ATCC13869*ΔcspBΔpbp1b*, respectively.

### Lysozyme sensitivity test of *C. glutamicum*

Lysozyme sensitivities of *C. glutamicum* ATCC13869, *ΔcspB*, *Δpbp1a*, *ΔcspBΔpbp1a*, *Δpbp1b*, and *ΔcspBΔpbp1b* mutants were evaluated by growth assay in LB liquid medium or plate assay, as described previously [51].

## Abbreviations

AP: Alkaline phosphatase; ATCC: American-type culture collection; CORYNEX®: *Corynebacterium glutamicum* protein expression system; Csp: Cell surface protein; Da: Dalton; Fab: Fragment antigen-binding; HC: Heavy chain; HMW: High molecular weight; IgG: Immunoglobulin G; LC: Light chain; LMW: Low molecular weight; OD: Optical density; PBP: Penicillin-binding protein; PCR: Polymerase chain reaction; PG: Peptidoglycan; PVDF: Polyvinylidene difluoride; RU: Resonance unit; S-layer: Surface layer; scFv: Single-chain variable fragment; SDS-PAGE: Sodium dodecyl sulfate polyacrylamide gel electrophoresis; Strain name/plasmid name: Denotes a plasmid-carrying strain.

## Competing interests

The authors declare that they have no competing interests.

## Authors’ contributions

YM designed and performed the Fab secretion and analyses, constructed the mutant strains, coordinated the work, and drafted the manuscript. HI designed the mutant strains and coordinated the work. YK and NMT performed the lysozyme sensitivity test. EAK and YAVY designed and constructed the mutant strains. MD assayed the antigen-binding activity of Fab. YK and MW supervised the work and reviewed the final manuscript. All authors read and approved the final manuscript.

## Supplementary Material

Additional file 1: Figure S1 Nucleotide sequence of the co-expression cassette of Fab(H+L) in pPKStrastFabHL, with the amino acid sequences given below. The sequence is presented in the 5′ to 3′ direction. The putative ribosome-binding site (RBS), the amino acid sequence of the CspA signal peptide, and restriction enzyme sites are boxed, underlined, and described in lower case, respectively. The sequences of HC and LC gene of the anti-HER2 Fab fragment are described in boldface. Click here for file

Additional file 2: Table S1 Bacterial strains and plasmids used in this study. Click here for file
