# Supplementary material for: Double mutation of cell wall proteins CspB and PBP1a increases secretion of the antibody Fab fragment from Corynebacterium glutamicum
Source: Microb Cell Fact. 2014 Apr 15;13:56. doi: 10.1186/1475-2859-13-56 (PMC4021378; doi:10.1186/1475-2859-13-56)
Supplement: Additional file 1: Figure S1 — Nucleotide sequence of the co-expression cassette of Fab(H+L) in pPKStrastFabHL, with the amino acid sequences given below. The sequence is presented in the 5′ to 3′ direction. The putative ribosome-binding site (RBS), the amino acid sequence of the CspA signal peptide, and restriction enzyme sites are boxed, underlined, and described in lower case, respectively. The sequences of HC and LC gene of the anti-HER2 Fab fragment are described in boldface. [file 1475-2859-13-56-S1.pdf]

Additional file 1: Figure S1

*Bam*HI
ggatccCAAATTCCTGTGAATTAGCTGATTTAGTACTTTTCGGAGGTGTCTATTCTTACCAAATCGTCAAGTTGTGGGTAGAGTCACCTG
AATATTAATTGCACCGCACGGGTGATATATGCTTATTTGCTCAAGTAGTTCGAGGTTAAGTGTATTTTAGGTGAACAAATTTCAGCTTCG
GGTAGAAGACTTTCGATGCGCTTCAGAGCTTCTATTGGGAAATCTGACACCACTTGATTAAATAGCCTACCCCCGAATTGGGGGATTGGT
CATTTTTTGCTGTGAAGGTAGTTTTGATGCATATGACCTGCGTTTATAAAGAAATGTAAACGTGATCAGATCGATATAAAAGAAACAGTT *cspB* promoter
TGTACTCAGGTTTGAAGCATTTTCTCCGATTCGCCTGGCAAAAATCTCAATTGTCGCTTACAGTTTTTCTCAACGACAGGCTGCTAAGCT
GCTAGTTCGGTGGCCTAGTGAGTGGCGTTTACTTGGATAAAAGTAATCCCATGTCGTGATCAGCCATTTTGGGTTGTTTCCATAGCAATC
CAAAGGTTTCGTCTTTCGATACCTATTCAAGGAGCCTTCGCCTCTATGAAACGCATGAAATCGCTGGCTGCGGCGCTCACCGTCGCTGGG
RBS M K R M K S L A A A L T V A G CspA signal
GCCATGCTGGCCGCACCTGTGGCAACGGCA**GAAGTGCAGCTGGTCGAGTCCGGCGGTGGCCTGGTGCAGCCTGGTGGCTCCCTGCGTCTC**
A M L A A P V A T A **E V Q L V E S G G G L V Q P G G S L R L**
TCCTGCGCAGCTTCCGGCTTCAACATCAAGGATACCTACATCCACTGGGTGCGTCAGGCACCAGGCAAGGGCCTGGAATGGGTCGCTCGT
S C A A S G F N I K D T Y I H W V R Q A P G K G L E W V A R
ATCTACCCTACCAACGGTTACACCCGCTACGCCGATTCCGTGAAGGGCCGTTTCACCATCTCCGCCGACACCTCCAAGAACACCGCGTAC
I Y P T N G Y T R Y A D S V K G R F T I S A D T S K N T A Y
CTGCAGATGAACTCCCTCCGCGCAGAGGACACCGCTGTCTACTACTGCTCCCGTTGGGGTGGCGATGGCTTCTACGCAATGGACTACTGG
L Q M N S L R A E D T A V Y Y C S R W G G D G F Y A M D Y W ***heavy chain***
GGTCAGGGCACCCTGGTTACCGTGTCCTCCGCATCCACCAAGGGTCCATCCGTCTTCCCGCTCGCTCCATCCTCCAAGTCCACCTCCGGT
G Q G T L V T V S S A S T K G P S V F P L A P S S K S T S G
GGCACCGCTGCTCTGGGTTGCCTGGTTAAGGATTACTTCCCAGAACCTGTCACCGTTTCCTGGAACTCCGGTGCTCTCACCTCCGGTGTC
G T A A L G C L V K D Y F P E P V T V S W N S G A L T S G V
CACACCTTCCCAGCTGTTCTCCAGTCCTCCGGTCTGTACTCCCTCTCCTCCGTGGTCACCGTTCCATCCTCCTCCCTGGGCACCCAGACC
H T F P A V L Q S S G L Y S L S S V V T V P S S S L G T Q T
TACATCTGCAACGTGAACCACAAGCCTTCCAACACCAAGGTTGATAAGAAGGTGGAGCCGAAGTCCTGCGACAAGACCCACACCTGCTAA
Y I C N V N H K P S N T K V D K K V E P K S C D K T H T C *
ggatcctctagaCAAATTCCTGTGAATTAGCTGATTTAGTACTTTTCGGAGGTGTCTATTCTTACCAAATCGTCAAGTTGTGGGTAGAGT
*Bam*HI *Xba*I
CACCTGAATATTAATTGCACCGCACGGGTGATATATGCTTATTTGCTCAAGTAGTTCGAGGTTAAGTGTATTTTAGGTGAACAAATTTCA
GCTTCGGGTAGAAGACTTTCGATGCGCTTCAGAGCTTCTATTGGGAAATCTGACACCACTTGATTAAATAGCCTACCCCCGAATTGGGGG
ATTGGTCATTTTTTGCTGTGAAGGTAGTTTTGATGCATATGACCTGCGTTTATAAAGAAATGTAAACGTGATCAGATCGATATAAAAGAA *cspB* promoter
ACAGTTTGTACTCAGGTTTGAAGCATTTTCTCCGATTCGCCTGGCAAAAATCTCAATTGTCGCTTACAGTTTTTCTCAACGACAGGCTGC
TAAGCTGCTAGTTCGGTGGCCTAGTGAGTGGCGTTTACTTGGATAAAAGTAATCCCATGTCGTGATCAGCCATTTTGGGTTGTTTCCATA
GCAATCCAAAGGTTTCGTCTTTCGATACCTATTCAAGGAGCCTTCGCCTCTATGAAACGCATGAAATCGCTGGCTGCGGCGCTCACCGTC
RBS M K R M K S L A A A L T V CspA signal
GCTGGGGCCATGCTGGCCGCACCTGTGGCAACGGCA**GATATCCAGATGACCCAGTCCCCATCCTCCCTGTCCGCATCCGTTGGTGATCGT**
A G A M L A A P V A T A **D I Q M T Q S P S S L S A S V G D R**
GTGACCATCACCTGCCGCGCTTCCCAGGATGTCAACACCGCAGTTGCGTGGTATCAGCAGAAGCCTGGCAAGGCACCGAAGCTGCTCATC
V T I T C R A S Q D V N T A V A W Y Q Q K P G K A P K L L I
TACTCCGCTTCCTTCCTCTACTCCGGTGTTCCATCCCGCTTCTCCGGCTCCCGTTCCGGCACCGATTTCACCCTGACCATCTCCTCCCTC
Y S A S F L Y S G V P S R F S G S R S G T D F T L T I S S L
CAGCCTGAAGACTTCGCAACCTACTACTGCCAGCAGCACTACACCACCCCACCTACCTTCGGCCAGGGCACCAAGGTCGAAATCAAGCGC
Q P E D F A T Y Y C Q Q H Y T T P P T F G Q G T K V E I K R ***light chain***
ACCGTTGCAGCTCCGTCCGTGTTCATCTTCCCGCCATCCGATGAGCAGCTGAAGTCCGGCACCGCTTCCGTGGTCTGCCTGCTCAACAAC
T V A A P S V F I F P P S D E Q L K S G T A S V V C L L N N
TTCTACCCACGTGAGGCCAAGGTGCAGTGGAAGGTCGACAACGCGCTGCAGTCCGGTAACTCCCAGGAATCCGTGACCGAGCAGGATTCC
F Y P R E A K V Q W K V D N A L Q S G N S Q E S V T E Q D S
AAGGACTCCACCTACTCCCTCTCCTCCACCCTGACCCTCTCCAAGGCAGACTACGAAAAGCACAAGGTCTACGCTTGCGAGGTTACCCAC
K D S T Y S L S S T L T L S K A D Y E K H K V Y A C E V T H
CAGGGCCTGTCCTCCCCAGTCACCAAGTCCTTCAACCGCGGTGAATGCTAAtctaga
Q G L S S P V T K S F N R G E C * *Xba*I

Figure S1 Nucleotide sequence of the co-expression cassette of Fab(H+L) in pPKStrastFabHL, with the amino acid sequences given below. The sequence is presented in the 5' to 3' direction. The putative ribosome-binding site (RBS), the amino acid sequence of the CspA signal peptide, and restriction enzyme sites are boxed, underlined, and described in lower case, respectively. The sequences of HC and LC gene of the anti-HER2 Fab fragment are described in boldfase.
