# Supplementary material for: Double mutation of cell wall proteins CspB and PBP1a increases secretion of the antibody Fab fragment from Corynebacterium glutamicum
Source: Microb Cell Fact. 2014 Apr 15;13:56. doi: 10.1186/1475-2859-13-56 (PMC4021378; doi:10.1186/1475-2859-13-56)
Supplement: Additional file 2: Table S1 — Bacterial strains and plasmids used in this study. [file 1475-2859-13-56-S2.pdf]

Additional file 2: Table S1 Bacterial strains and plasmids used in this study

Bacterial strains and plasmids	Relevant characteristics	Source or references
Escherichia coli		
JM109	*recA1 endA1 gyrA96 thi-1 hsdR17(rK- mK+) e14- (mcrA-) supE44 relA1Δ(lac-proAB)/F'[traD36 proAB+ lac Iq lacZΔM15]*	Takara Bio, Tokyo, Japan
Corynebacterium glutamicum		
ATCC13869	Wild type	ATCC
AJ12036	ATCC13869 *N*-methyl-*N'*-nitro-*N*-nitrosoguanidine mutant	[40]
YDK010	AJ12036 *ΔcspB*	[40]
YDK010*Δpbp1a*	AJ12036 *ΔcspBΔpbp1a*	This study
YDK010*Δpbp1b*	AJ12036 *ΔcspBΔpbp1b*	This study
ATCC13869*ΔcspB*	*ΔcspB*	[41]
ATCC13869*Δpbp1a*	*Δpbp1a*	This study
ATCC13869*Δpbp1b*	*Δpbp1b*	This study
ATCC13869*ΔcspBΔpbp1a*	*ΔcspBΔpbp1a*	This study
ATCC13869*ΔcspBΔpbp1b*	*ΔcspBΔpbp1b*	This study
Plasmids		
pPK4	*E. coli-C. glutamicum* shuttle vector, Km^r	[18]
pPKStrastFabHL	pPK4 carrying the heavy chain and the light chain genes of the Fab region of "trastuzumab" fused with the signal peptide of CspA from *C. ammoniagenes* under control of the *cspB* promoter from *C. glutamicum*, respectively	This study
pBS5T	*E. coli-C. glutamicum* shuttle vector, temperature-sensitive origin, Km^r, *sacB* (*B. subtilis*)	[49]
pBS5TΔpbp1a	pBS5T carrying the flanking regions of the *pbp1a* gene	This study
pBS5TΔpbp1b	pBS5T carrying the flanking regions of the *pbp1b* gene	This study
